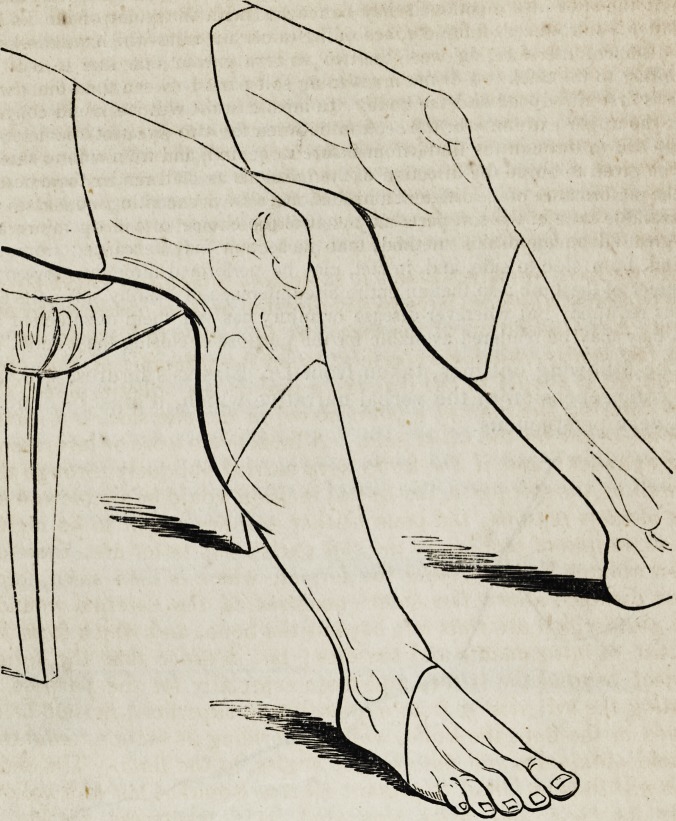# The Oblique Section.—A New Method of Amputation, to Which Is Added, an Enquiry into Other Circumstances Respecting Amputation in General

**Published:** 1839-07

**Authors:** 


### Art. IX.

*Der Schrägschnitt, eine neue Amputationsmethode, nebst Erörterungen anderer, die Amputationen betreffender Gegenstände.* Von Ernest Blasius, m.d., Professor der Chirurgie zu Halle, &c.—*Berlin*, 1838. 4to, pp. 70.

*The Oblique Section.—A new Method of Amputation, to which is added, an Enquiry into other Circumstances respecting Amputation in general.* By Ernest Blasius, Doctor of Medicine and Surgery, &c. &c. &c., with Six Plates.—*Berlin*, 1838. 4to, pp. 70.

In the essay before us, which extends through seventy pages of quarto, the learned Doctor has, with much labour and research, presented to his readers all the different methods of amputation which have been recommended and practised in the times of modern surgery; he has compared and contrasted them in all their bearings, by enumerating their respective advantages, and placing against the latter the objections to which they are obnoxious. The result of his investigations and experience has led him to adopt the "oblique section," as combining all the advantages on the one hand, while it eschews the evils on the other. In an operation which, however modified, must always consist in cutting through the soft parts and sawing off the bone, in such a manner as to leave a covered stump, nothing essentially new in principle can be expected; and the chief novelty of our author's method will be found in the peculiar shape of his instrument and the manner in which it is used. Both the one and the other are described with more than German minuteness; and we shall endeavour to make them intelligible to our readers by *woodcuts*, and by extracting from the book that portion which immediately bears on the operation and the precise mode of performing it, first apologizing to the Doctor for having occasionally departed from the text in order to curtail its prolixity and rectify its involutions. We fear, however, that most of our English surgeons would shrink from the quaint-looking knife which is here described and delineated, requiring the use of both hands for its guidance; and we cannot but imagine that its introduction into a London operating theatre would excite as much surprise as the sight of a regiment of soldiers armed with the six-foot double-handed broad swords, with which our ancestors were wont to deal their doughty blows.

"The characteristic feature of the oblique section," says Dr. Blasius, "is this. The soft parts are divided by two incisions, both of which have a double slanting direction, inasmuch as they are oblique to the transverse as well as to the long axis of the limb. These incisions are carried round the bone in such a manner that, when the latter has been sawn through, it will be found to occupy the deepest part of the wound, while the section of the limb, when the operation is completed, will be found to represent an oval figure, one extremity of which is situated near to the part where the saw has been applied, while the other terminates at a considerable distance beyond it. The stump, before it is closed up, resembles a funnel, from which the whole of the base, and the greater part of one side, has been removed by an oblique section; or perhaps it may still more aptly be compared to a shallow paper-cone, with its apex encircling the bone; and in the same way as we shut up a paper-cone, by folding the lappet over the top, so does the surgeon close his stump, by bringing up the lower angle of the flap, and adapting it to the corresponding fissure at the upper part of the wound." (p. 7.)

For the performance of this operation, Dr. B. uses a strong knife, five inches eight lines long in the blade, four inches long in the handle, which latter is ten lines in width and six in thickness. The blade measures eight lines across near the handle, and is single edged to within about two inches to its extremity; the remaining part is double edged, and the back is carried out so as to increase the width to thirteen lines. The blade terminates in a convex point, while the cutting edge at the back ceases suddenly, and presents a broad concave surface, adapted to afford a secure rest for the finger.

1

2

1. Back view of the knife. 2. Side view of the knife.

'A knife of the above dimensions may be used for removing any of the larger limbs which do not exceed the average size. For amputations below the knee in children, a smaller knife of similar shape is employed; and in certain cases, where the soft parts are few and the limbs very small, a convex scalpel will answer the purpose. It may be advisable, before commencing the operation, to trace out on the limb the track of the incisions, or at any rate to mark with accuracy the points, above and below, in which the two incisions will meet, and which constitute the two extremities of the oval surface, represented by the wound after the removal of the limb. It is not essential, for the subsequent closing up of the wound, that the two extremities of the oval should be exactly opposite, or, in other words, that an imaginary line, drawn between them, should pierce the centre of the limb; and, in fact, when there is a preponderance of soft parts on one side, it may frequently be desirable to make the incisions of different lengths, so that the longest may extend along the thickest side of the limb. By this arrangement, the edges of the flap will be more readily adjusted and brought into contact when the wound is closed. Too great a disparity, however, in the length of the cuts, throws difficulties in the way of their completion. The distance between the two extremities of the oval section, measuring along the length of the limb, must amount to from one half to two thirds of its transverse diameter, at the part where the removal takes place, the variation depending upon the greater or less degree of yielding, of which the skin and muscles may be susceptible. (Thus, if the incisions commence on the fore and terminate on the back part of the limb, the terminations would be opposite to a point distant from the commencement, from half to two thirds the diameter of the limb at the part where the bone is sawn through.) The upper extremity of the oval should lie somewhat below the spot where the bone is cut through, indeed, just so much below, that from it the spot just mentioned may be reached with the point of the knife, when the latter is introduced obliquely, according to certain rules which will be laid down. The distance, therefore, between the two will vary from one line to half an inch, according to the thickness of the intervening soft parts between the points where the knife is first introduced and the bone. In most cases it will be found convenient to place the upper extremity of the section between the two greatest diameters, which can be drawn through the limb, from before to behind, and from side to side. Rules will be given to guide the direction of the incisions as shall render them best adapted for the performance of the different amputations, always excepting those cases where an unfavorable state of the soft parts may control the choice of the operator; and it is one great advantage of this method, that the section may be carried from before to behind, from side to side, and, in fact, may be performed through every conceivable diameter of the limb; so that an entire circumference of healthy soft parts are by no means required, but wherever disease or injury has left them in sufficient quantity, then they may be rendered available for the purpose of closing the wound."

The following outlines, taken from Dr. Blasius's figures, will facilitate the comprehension of the verbal narrative which, it must be confessed, is not very perspicuous.

"After the usual precautions for suspending hemorrhage have been taken, the operation is performed in the following manner: Two assistants are required, whose business it is to draw upwards the integuments and soft parts, and at the same time to press them firmly around the bone, just above the part where the section is to be made. The operator, standing on the right side of the limb, takes the knife in his right hand, his thumb resting in the cavity cut out to receive it at the heel of the blade, and carries the instrument under the limb, so as to reach with its point the upper angle or commencement of the section previously marked out. Holding the knife so that both the length and breadth of its blade shall be directed obliquely towards the limb, he lays his left fore-finger on the rest which is cut out on the back of the blade, and by the pressure of his finger alone pushes the point down to the bone. He then carries the edge along the indicated oblique line, keeping the convex point as closely as possible in contact with the bone, and on reaching the lower angle or point where the flap is to terminate, directs the knife so as to bring it into the second line of incision on the side of the limb opposite to that on which he has just cut. While he is thus changing the direction of the knife, a manœuvre accomplished without the least difficulty, he alters his grasp of the handle, by bringing the end of his fore-finger into the rest at the heel of the blade previously occupied by his thumb, and at the same time places his left thumb on the rest at the back of the blade. The point is again pushed to the bone by the pressure of the left thumb, and the knife is carried upwards, by observing the same rules which regulated the first incision, until it reaches the point where the operation commenced. As soon as this is accomplished, the operator seizes the lower angle of the flap with his left hand, draws it back, and by a few strokes of his knife separates the remaining soft parts from the bone, thus laying bare the latter as high as where the saw is to be applied. In limbs containing two bones a catlin must be used to separate the deep-seated muscles; but the operation is equally applicable to the removal of the thigh, the leg, the upper arm, or the fore-arm. A linen retractor is applied to hold back the flap, while the periosteum is divided and the bone sawn through in the usual manner. The wound is closed by bringing up the flap so as to unite the upper and lower angles of the section. The lower half of the oval figure is in fact folded upon the upper, so that while the surfaces of the two are brought into close apposition, their margins will be found to correspond with each other." (pp. 8-12.)

As we have ourselves found it extremely difficult to follow the doctor's descriptions (which we have partly divested of their obscurity by adopting a very free translation of the text), we beg leave to state to our readers, in a few words, what we consider to be the principle of this operation. Its object is simply to cut through a limb in an oblique plane, the incision entering the limb at a point above on the one side, and leaving it at a point below on the other side. A limb bears more or less resemblance to a cylinder; and if the knife were carried obliquely through the bone as well as the soft parts, the line of incision would be simple and straight. For obvious reasons, the bone neither can nor ought to be included in the same line of section as the soft parts; the latter are, therefore, cut in an oblique line all round the former, which is then sawn through at some distance above the commencement of the external wound. The soft parts which are thus left beyond the bone, and which form the flap, consist of integuments and muscles; but in order that the former may project beyond the latter, and more especially for the purpose of preventing the evil arising from a quantity of superficial muscle being contained in the flap, the knife, while performing its section round the bone, is held obliquely, and not at right angles, to the limb. The end of the limb will thus be found to present all that would be left of a hollow cone, after its apex had been truncated by a transverse section, and its base, together with the greater portion of one side, removed by an oblique section. The end of the bone lies in the bottom of the wound, forming the truncated extremity of the cone.

There is nothing essentially new in this operation. In principle, if not in detail, it has often been had recourse to, in cases where, either from injury to the soft parts or from disease, a deficiency of integument, necessary for circular or lateral flaps, has compelled the operator to seek a covering for his stump from whatever source he could obtain it. Compulsion rather than choice has hitherto led to its adoption; but by some surgeons it has been preferred to the more ordinary sections; and time alone can show whether the recommendation of Dr. Blasius, founded on considerable experience of its efficacy, may not render the oblique section the more common mode of amputation. The well-known method of saving a flap from the calf of the leg, so frequently had recourse to where a circular flap cannot be obtained, differs but slightly, if we except the detail of the operation, from the oblique section so elaborately described by our author.

Our own experience and observation has convinced us that the successful issue of an amputation depends mainly on the observance of two or three rules which are applicable to all methods of performing the operation. 1. It is essential to have sufficient integumental flap (which may or may not include a portion of the superficial muscles adhering to it,) to cover the stump. The size of this flap should be adapted as accurately as possible to the surface it is intended to cover; if too large, it will bag and hang loosely, and be constantly shifting its position; if too scanty, it must be strained into apposition. In either case no adhesion will be obtained. 2. The section necessary for obtaining a flap should be made by as few cuts and these executed in as clean and decisive a manner as possible; the knife should never be employed twice to effect what may be obtained by one application. Where the flap is intended to include muscle as well as integument, the knife should always, if possible, be carried to the bone in one sweep. Unless this character of decision is given to the section, the same line of animal fibre, be it muscle, cellular membrane, nerve, or vessel, necessarily becomes divided in more than one point, and the surface is jagged and irregular, instead of being smooth and clean. 3. When the flap has been completed, the deep-seated muscles should be carefully and skilfully separated from the bone up to a certain distance,—in fact, to such an extent as shall furnish a quantity of muscular fibre, thus separated, sufficient to cover the end of the bone, to form a sort of cushion before it, and separate it from the integumental flap. This constitutes in our view a most important step in the operation—one often neglected, because it requires time and mars the *eclât* which attends the rapid removal of a limb; but the neglect is the more reprehensible, inasmuch as the sin of omission incurred by the surgeon is sure to be visited on the patient, and in too many cases the thirty seconds saved in the operation by the former are afterwards paid for by the latter at the price of thirty days of protracted suffering and confinement. We think that the importance of having a cushion of deep-seated muscle to cover the bone, although constantly urged, has not been sufficiently attended to in practice, perhaps because its precise utility has not been properly understood or explained. Indeed, a muscular covering to a stump has been altogether deprecated, as being liable to subsequent contraction and exposure of the bone. To a certain extent this objection obtains, as far as the superficial or long muscles are concerned, but has certainly no reference to the deep-seated muscles which derive their origin from the bone which they immediately surround. When the superficial or long muscles (for the terms are nearly synonymous) are divided, if they contract, they are drawn towards their remaining fixed point, and, consequently, recede from the stump. Not so the deep-seated muscular fibre, which, on being separated from the bone, is at once deprived of both its fixed points, and therefore no longer retains its power of altering its position during contraction. It is immediately converted into a mass of loose muscular tissue, having no attachment whatever, save its cellular and vascular connexion to the surrounding soft parts; and the contraction of its fibres being no longer capable of influencing its position, it falls before the end of the bone and remains for ever in that situation : it therefore forms a permanent cushion composed of a material the very best that could be chosen for covering the bone.

The remaining portion of Dr. Blasius's essay is partly occupied by a comparison of his own operation with all the other methods of removing limbs which have ever been devised or executed. Then follows a minutely detailed account of the way in which each particular limb of the body should be amputated, illustrated by plates, in which the various lines of incision are marked out on the arms and legs. The whole concludes with copious remarks on amputations in general, the prognosis to be formed or the result which may be anticipated when we have recourse to the operation for different forms of disease, the after-treatment, more particularly with reference to the management of the stump, &c.

---

## Figures and Tables

**Figure f1:**
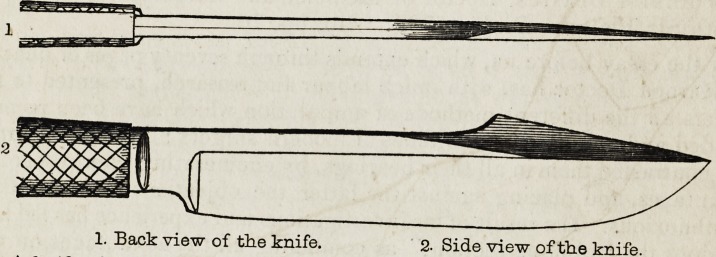


**Figure f2:**
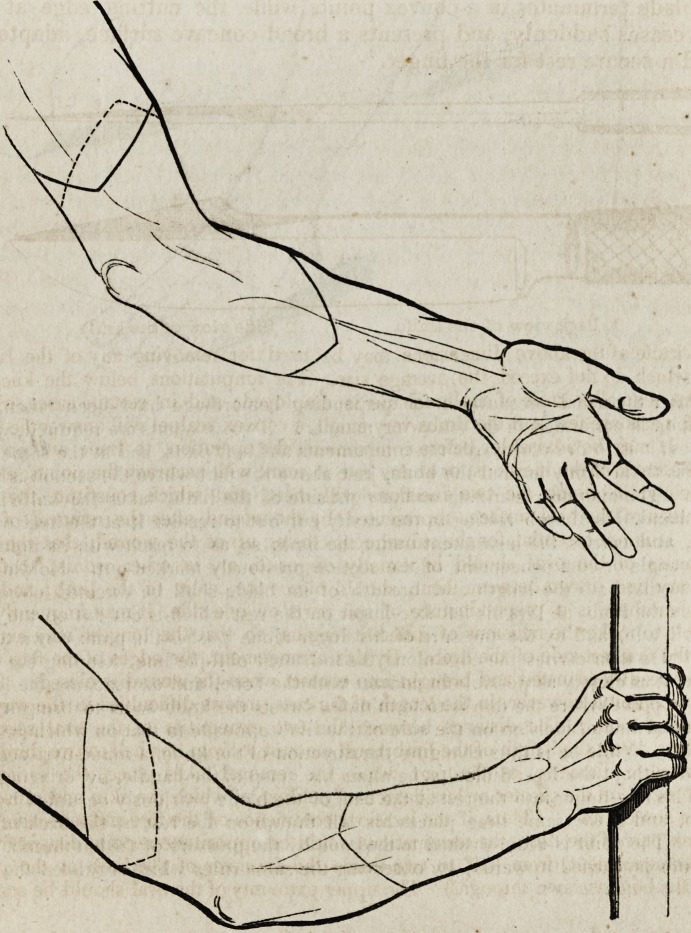


**Figure f3:**